# Functional assessments for decision-making regarding return to sports following ACL reconstruction. Part I: development of a new test battery

**DOI:** 10.1007/s00167-015-3529-4

**Published:** 2015-02-15

**Authors:** Carolin Hildebrandt, Lisa Müller, Barbara Zisch, Reinhard Huber, Christian Fink, Christian Raschner

**Affiliations:** 1 Department of Sport Science, University of Innsbruck, Fürstenweg 185, 6020 Innsbruck, Austria; 2 Sports Physiotherapy Mag. R. Huber, Innsbruck, Austria; 3 Sportsclinic Austria, Innsbruck, Austria

**Keywords:** Return to sports, Test battery, Normative data, Limb symmetry index, Anterior cruciate ligament injury

## Abstract

**Purpose:**

Return to activity remains the most common concern following an injury. To facilitate the decision regarding a patient’s return to sport, we developed a standardized and easy-to-use test battery to enable an objective evaluation of knee function.

**Methods:**

The test battery consisted of seven functional tests: the two-leg stability test, one-leg stability test (OL-ST), two-leg countermovement jump (CMJ), one-leg CMJ (OL-CMJ), plyometric jumps, speedy test and quick feet test. For each test, the reliability was determined based on the intraclass correlation coefficient. For all one-leg tests, the limb symmetry index (LSI) was calculated.

**Results:**

All tests showed a moderate-to-high reliability. Normative data from 434 participants were included in the analysis. The subjects were categorized according to age as follows: children (10–14 years), youth (15–19 years), young adults (20–29 years) and adults (30–50 years). The establishment of the functional test values allowed the classification into five normative categories. The LSI for the OL-ST (98 %) indicated a better performance of the non-dominant leg. In contrast, high LSI values were found for the OL-CMJ (124 %), indicating a better performance of the dominant leg.

**Conclusion:**

Each test was found to be reliable and simple to perform. The better performance of the non-dominant leg in stability tasks must be considered when interpreting side-to-side differences. The established norm data from healthy individuals of each test battery represents an important basis for a clinical setting. Test results from an ACL-reconstructed patient should be at least classified as a functionally average outcome to support a safe return to sports.

**Level of evidence:**

IV.

## Introduction

A large amount of scientific research is directed towards anterior cruciate ligament (ACL) injuries. However, recent epidemiological studies have demonstrated a continued high incidence of ACL injuries despite the implementation of intervention programs [3]. As these injuries remain a significant problem, research directed at rehabilitation is needed. An injured athlete is under pressure to return to competition as soon as possible and there is significant interest among sports medicine professionals in identifying an adequate indicator of safe return to activity [12, 17, 29, 30]. Between 43 and 92 % of athletes return to their sports within 6–12 months following ACL reconstruction [14]. Another important fact to note is the unacceptably high re-injury rate of reconstructed ACLs [20]. In addition to clinical examinations such as Lysholm’s score and the Cincinnati Knee Ligament Rating scale, functional performance tests are commonly used to predict athlete’s abilities to participate in sports [1]. Some clinicians use isolated functional tests, such as hop tests [5, 15, 26, 27], but these tests have been criticized for not being suitable for the evaluation of sufficient functional capability in ACL-reconstructed patients [23]. Muscle strength tests were used to identify differences between injured and non-injured legs. The results showed that the patients reported poor results with respect to knee function during activities and a high fear of re-injury despite sufficient muscle strength capacity in both legs [2, 4, 25]. This observation demonstrated that muscle strength tests are not sensitive enough to distinguish between the functional differences between injured and non-injured legs and “time since-surgery criteria” are unsuitable to guide safe return to sport [19]. Next to single functional test, there are a few studies that examined test batteries in ACL-reconstructed patients as a measure of function [9, 10, 22], but they did not provide information necessary for determining readiness for return to sport [23]. Most of these tests in a clinical setting were applied as an outcome measure based on scores obtained from the ACL-reconstructed patient. In order to classify whether the test result can be considered as a functionally average outcome, norm data of uninjured subjects are crucial for the judgement. To date there is no study that provides data of functional measurements in a healthy population as a reference. Furthermore, most of these studies were conducted at least 1 year postoperatively; investigations performed during the early rehabilitation period (3–6 months) are missing. Evaluations of progress over time, which are necessary to guarantee a confident postoperative return to training and competition within an appropriate time frame, are still a major concern. There is a need to identify complex, persistent functional deficits related to the initial ACL reconstruction. To our knowledge, there is only one test battery that combines various subtests to broadly address sport-specific requirements [22]. The large-scale setting design and lab-based materials in this study may limit a regular application. To support decision-making, we therefore developed and evaluated a standardized test protocol combining various subtests (power, speed, agility, coordination, unilateral cutting and side-to-side movements) that require no sophisticated equipment and can be used multiple times within the rehabilitation period. We classified test results into five different performance categories and established potential limb asymmetries in a healthy population. We hypothesized that the test battery represents the basis for a clinical setting by objectively determine the right time point for a safe return to sport post ACL reconstruction. We further hypothesized that healthy individuals did not exhibit relevant limb asymmetries independent of the test requirements, age and gender. The long-term aim is the clinical application of the test battery. Test results from an ACL-reconstructed patient should be at least classified as a functionally good outcome to support a safe return to sports and to decrease the likelihood of re-injury by returning to sport too quickly.

## Materials and methods

### Reliability

For reliability testing, data from twenty eight (thirteen female, fifteen male) healthy subjects (age 24.1 ± 2.5 years) were obtained for each test. Exclusion criteria included previous knee, hip or ankle injuries as well as current pain somewhere of the musculoskeletal system. Before testing, each subject completed a 15-min warm-up at a submaximal level on a bike cycle, followed by 5 min of individual dynamic stretching and jumping. The order of the test stations were similar to the subsequent recommendations of the present test battery, starting with TL-ST and OL-ST on a MFT Challenge Disc, followed by all jumping test with the Myotester (TL-CMJ, OL-CMJ, TL-PY), the OL-SY and finally the TL-QFT. On each test station, each subject performed two trials for familiarization. After a rest of 3 min, the test was performed until three successful and valid trials were made. To ensure adequate recovery between tests, each subject got 5 min of rest between the test stations. Test procedure and equipment were the same as used for the evaluation of normative data. The test leader of each station was the same between test and re-test. Verbal encouragement and instructions were standardized. There was a 5-day interval between the tests, and subjects were asked to avoid strenuous physical activity 3 days before testing.

### Normative data

Over the 2-year period of data collection, more than 450 subjects were randomly selected to participate in this study. The participants were not engaged in competitive sports and had no experience with activities similar to the exercises used in the test battery. Prior to testing, the procedure, including possible risks and benefits, was explained to the subjects. They (or, if under 18 years of age, their legal guardians) were asked to read and sign the informed consent document. Exclusion criteria included previous knee, hip or ankle injuries. Additionally, none of the subjects exhibited evidence of acute pain. We only included subjects who performed all tests in the specified order. In total, data from 434 subjects were included in the study. For each subject, age, height, weight, body mass index (BMI) and the dominant leg were recorded (Table 1). Based on the study by Coren et al. [11] who used similar survey items to determine a person’s dominant leg, we asked the following question: “Which leg do you use to push a ball as strongly as possible?” Based to the result, each participant had to perform the following two tests, in which the first leg used was defined as the dominant leg.

1. Climb a stairwalker
2. Participants were prodded into action by us giving them a slight push

**Table 1:** Descriptive characteristics of healthy subjects in all age groups

	Age (years)	Height (m)	Weight (kg)	BMI (index)	Dominant leg (%)
Children (10–14 years)					
Male (*n* = 57)	10.9 (±0.7)	1.47 (±0.06)	39.8 (±8.8)	18.4 (±3.4)	98-Right
Female (*n* = 50)	11.7 (±0.7)	1.52 (±0.08)	43.3 (±9.5)	18.8 (±4.0)	96-Right
Youth (15–19 years)					
Male (*n* = 57)	16.2 (±0.8)	1.77 (±0.07)	67.8 (±10.8)	21.6 (±3.1)	96-Right
Female (*n* = 70)	16.1 (±1.1)	1.67 (±0.06)	59.8 (±9.8)	21.4 (±3.5)	97-Right
Young adults (20–29 years)					
Male (*n* = 51)	24.8 (±2.6)	1.79 (±0.06)	74.8 (±6.3)	23.3 (±1.7)	85-Right
Female (*n* = 51)	23.6 (±2.5)	1.65 (±0.06)	59.5 (±7.1)	20.9 (±1.9)	69-Right
Adults (30–50 years)					
Male (*n* = 51)	38.2 (±7.3)	1.79 (±0.05)	77.3 (±7.2)	24.1 (±1.8)	86-Right
Female (*n* = 47)	37.5 (±6.1)	1.69 (±0.06)	61.3 (±7.0)	21.5 (±2.1)	75-Right

All values are expressed as means (±SD)

Before testing, the subjects completed a warm-up consisting of 10 min of stationary cycling (female 1.5 W/kg, male 2.0 W/kg). The test battery included seven different tests conducted in a systematic order. For each test, two practice trials, followed by three approved trials, were performed. Each trial with the best performance was selected for analysis. One minute of rest was included between each trial. Table 2 provides an overview of the test descriptions and variables measured. For the one-leg tests, the previously defined dominant leg was the first leg to be tested. All procedures were reviewed and approved (ID-Number 022014) by the Board of Ethical Questions in Science of the University of Innsbruck.

**Table 2:** Description of the test battery

Test	Description
Stability test	To assess postural control, tests were performed on an *MFT Challenge Disc* (*TST Trendsport, Grosshöflein, Austria*) connected to a PC. The disc is free to move in all directions. While balancing on the disc, Coordi software provides instant feedback about the position of the disc. To avoid the influence of different shoe types, all trials were performed without shoes. Subjects were instructed to stand in the centre with their arms at their sides *Variable* level of stability [Index] *Two*-*leg stability test* (*TL*-*ST*) subjects had to stand with both legs on the disc while maintaining their balance for 30 s. Data collection was immediately stopped in the case of a loss of balance *One*-*leg stability test* (*OL*-*ST*) based on the two-leg test results, the test was performed with one leg. The subject was not allowed to stabilize the raised leg against the plate or standing leg
Jumping test	All jump tests were performed using *Myotest* (*Myotest S.A., Sion, Switzerland*) equipment. The subjects carried a belt around their hips, and the Myotester was placed above the greater trochanter of the hip. Before jumping, the subjects had to stand in an upright and still position *Variables* jump height (cm), Power (W/kg), ground contact time (ms), reactivity (mm/ms) *Two*-*leg countermovement jump* (*TL*-*CMJ*) a sound signal from the Myotester announced the start of the jump. From an upright position, the subjects quickly bent their knees and then immediately jumped upward, attempting to maximize their height. During this hop, arms were placed on the hips *One*-*leg countermovement jump* (*OL*-*CMJ*) this was similar to the two-leg test, but this test was performed with one leg *Plyometric jump* (*TL*-*PJ*) the subject had to perform three consecutive two-leg jumps, focusing on a maximum jump height and a fast ground contact time. Arms could be used to assist with the jump
	*Speedy jump* (*OL*-*SY*) the Speedy Basic Jump Set *(TST Trendsport, Grosshöflein, Austria)* was used to create the jump coordination path (right panel). The subjects performed one-footed jumps through the course of red (forward–backward–forward jumps) and blue (sideway jumps) hurdles, completing 16 jumps. This had to be performed as quickly as possible by jumping on one leg without a rest between the hurdles. Twisting of the hip was not allowed, and the test was immediately stopped when the raised leg touched the ground or the subject had direct contact with the speedy basic jump hurdles. Time was measured using two stopwatches beginning as soon as the subject started to jump and ending when he/she reached the finish line with one leg. The mean value was recorded for each jump *Variables* time (s)
	*Quick feet test* (*TL*-*QFT*) again, the Speedy Basic Jump Set *(TST Trendsport, Grosshöflein, Austria)* was used for the quick feet test as displayed in the picture. The subject had to step in and out with one foot after the other until 15 repetitions were completed. One repetition was finished when the starting leg returned to its initial position. The test was stopped if the subject reversed the order of the steps. Arms could be used to maintain balance, and stepping on the speedy pole was not allowed *Variables* time (s)

### Statistical analysis

#### Reliability

To assess the test–retest reliability, measurements from the first testing day were correlated with measurements from the same tester’s second testing day. Test–retest reliability was determined using the intraclass correlation coefficient (ICC 1/1) in the one-way random effects model. This correlation value reflects the percentage of variance between the day-to-day measurements.

#### Normative data

To enable an objective evaluation, normative data from healthy subjects were established for each test. To generate these data, the participants were categorized according to age and gender. The best test value for each subject was selected for analysis. The mean values in each category were calculated and were defined as “average”. To establish further categories, half of the standard deviation was both added to the mean value (“good”, “very good”) and subtracted from it (“weak”, “very weak”). In total, seven categories were generated for each test based on gender and age. In addition, we calculated the lower limb symmetry index (LSI) for the one-leg stability test and countermovement jump to determine whether there was a side-to-side difference between the dominant- and non-dominant legs. The LSI is defined as the ratio of the dominant leg score and the contralateral leg score expressed as a percentage (dominant/non-dominant × 100 = LSI) [8, 24]. For the one-leg tests, we also defined specificity, expressed as the number of subjects classified as having a normal LSI divided by the total number of subjects. According to previous studies, a normal LSI was defined as ≤10 %. All inferential statistical analyses were conducted using SPSS statistical software 17 for Windows. Functional performance test data of 434 participants were included in the analysis.

## Results

Table 1 presents the descriptive characteristics and the number of participants in all age groups. The mean BMI values showed a normal average weight for each age category. The right leg was found to be the dominant leg for most of the participants.

### Reliability

Data from 28 participants were included in the test–retest reliability study. The ICC indicated a high reproducibility for the TL-CMJ and a moderate reproducibility for the TL-ST. All other tests showed good test–retest reliabilities. Table 3 presents the ICC values for all the functional tests.

**Table 3:** ICC values of the functional tests

Functional test	Unit	Leg	Test	Retest	ICC
TL-ST	Level		2.60 (±0.47)	2.49 (±0.36)	0.688
OL-ST	Level	domL	2.51 (±0.50)	2.27 (±0.49)	0.763
		n-domL	2.40 (±0.45)	2.31 (±0.46)	0.819
TL-CMJ	Height (m)		38.1 (±0.7)	37.6 (±0.6)	0.921
	Power (W/kg)		45.7 (±7.8)	47.2 (±7.7)	0.889
OL-CMJ	Height (m)	domL	23.6 (±4.5)	23.9 (±4.1)	0.897
	Power (W/kg)	domL	36.2 (±8.1)	36.0 (±7.8)	0.822
	Height (m)	n-domL	20.2 (±5.2)	21.1 (±4.9)	0.778
	Power (W/kg)	n-domL	32.8 (±7.8)	33.5 (±8.5)	0.814
TL-PY	Index		2.0 (±0.4)	1.9 (±0.5)	0.838
OL-S	Time (s)	domL	6.3 (±0.8)	6.1 (±0.7)	0.792
		n-domL	6.4 (±0.9)	6.3 (±0.9)	0.825
TL-Q	Time (s)		8.7 (±1.3)	8.4 (±1.0)	0.803

Values are expressed as means (±SD)

### Normative data

**Fig. 1:**
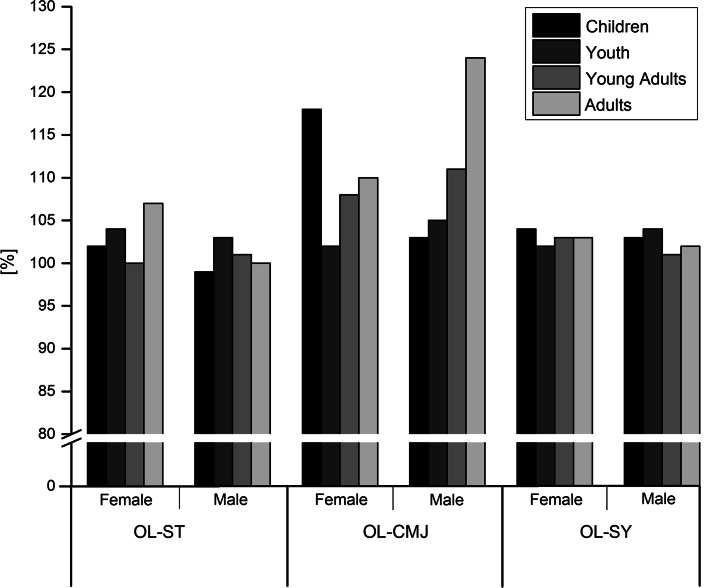
Limb symmetry index of all one-leg tests

For the data analyses, the subjects were categorized into the following 4 different groups according to their age: children (10–14 years), youth (15–19 years), young adults (20–29 years) and adults (30–50 years). The establishment of the functional test values allowed for the creation of five normative categories. For each test, normative data were defined according to age group and gender. As an example, Table 4 represents the five categories of normative data for the one-leg stability test. For all one-leg tests, reference values were obtained for the dominant and non-dominant legs. All categories were colour-coordinated to facilitate classification as very good (dark green), good (light green), average (yellow), weak (orange) and very weak (red). Figure 1 indicates the LSI of all one-leg tests. Results for the OL-ST revealed a score of 98 % in male children, indicating a better performance (less time) for the non-dominant leg. In the female young adult and male adult groups, no differences could be found between the dominant and non-dominant legs (LSI 100 %). Only female adults showed better performance in the dominant leg compared with the non-dominant leg, with an LSI of 107 %. Similar results, with small performance differences between the two legs, were reported for the OL-ST, where the LSI ranged from 101 to 104 %. In contrast, high values of the LSI were found for the OL-CMJ, indicating a better performance of the dominant leg (better jump height). Female children (118 %) and male adults (124 %), specifically, exhibited large side-to-side differences.

**Table 4:** Examples of normative values for the one-leg stability test for the five categories

NORMDATA		NORMDATA	
Female; non-dominant leg		Female; dominant leg	
Level (index)	Description	Level (index)	Description
≤1.74	Very good	≤1.76	Very good
1.75–2.05	Good	1.77–2.00	Good
2.06–2.90	Average	2.01–2.72	Average
3.00–3.30	Weak	2.73–2.96	Weak
≥3.31	Very weak	≥2.97	Very weak

NORMDATA		NORMDATA	
Male; non-dominant leg		Female; dominant leg	
Level (index)	Description	Level (index)	Description
≤1.98	Very good	≤2.00	Very good
1.99–2.31	Good	2.01–2.30	Good
2.32–3.09	Average	2.31–3.19	Average
3.30–3.62	Weak	3.20–3.49	Weak
≥3.63	Very weak	≥3.50	Very weak

Table 5 shows the specificity of the three one-leg tests. The one-leg countermovement jump had the lowest specificity in all age groups, ranging from 40 % (male adults) to 63 % (male children). Independent of gender and age, the one-leg stability test showed a mean specificity ranging from 66 % (male adults) to 78 % (male children). The one-leg speedy test had the highest specificity in all age categories. The test reached a specificity of greater than 90 % in female and male children, as well as in female and male young adults.

**Table 5:** Specificity results for all one-leg tests

Age category	Gender	Specificity (%)		
		OL-ST	OL-CMJ	OL-SY
Children	Female	71	41	93
	Male	78	63	95
Youth	Female	68	53	88
	Male	67	59	90
Young adults	Female	76	51	92
	Male	74	47	93
Adults	Female	71	45	86
	Male	66	40	87

## Discussion

The most important finding of the present study was the evidence of limb asymmetries of more than 10 % in healthy individuals during hop performance. In one-leg hop testing, the LSI has been suggested as an indicator of normal or abnormal side-to-side differences. The European Board of Sports Rehabilitation recommends hop performance differences of ≤10 % between the injured and non-injured legs for competitive sports; however, to date, evidence-based data that can be used to distinguish between normal and abnormal performances are limited. In the present study, one maximum one-leg hop test (OL-CMJ) and one endurance hop test (OL-SY) were used to increase the opportunity to detect differences in hop performances between legs. The present study demonstrated an LSI of up to 124 % for the OL-CMJ in healthy male adults. The countermovement jump with a vertical jump is regarded as a symmetrical simultaneous motion. There are conflicting results regarding the functional differences between dominant and non-dominant legs. Kimura et al. [18] reported no equivalent muscular output between the right and left legs in vertical jumps. In contrast, a study by Abrams et al. [1] demonstrated an LSI of >90 % in healthy subjects. These findings are contrary to our results. In Abrams’ study, the subjects were allowed to use their hands, while subjects in the present study had to keep their hands on their hips. If subjects are restricted from using additional centrifugal mass, the examination of the isolated jump power is dominant, which may explain the higher side-to-side differences. To further investigate the LSI for the one-leg tests, we calculated its specificity, which indicated the probability that the one-leg tests would reveal a normal LSI for healthy subjects. Specificities for the OL-CMJ ranged from 41 to 63 % and were lower than those derived from the one-leg stability test (66–78 %) and the one-leg speedy test (86–95 %). These findings are similar to those in the study of Barber et al. [6], where the specificity ranged from 48 to 90 %, and many of the healthy subjects performed the vertical jump outside of the normal LSI. Four of the seven functional tests were hop tests. According to the literature, jump performance seems to be an important outcome factor for a safe return to sport following injury [13]. Ardern et al. [4] stated that patients with good hop performances were more likely to return to sport compared with those with poor performances. We also generated normative data for the two-leg plyometric jump to assess the bounce quality and intermuscular coordination of both legs. The plyometric jump demands a consecutive output of instantaneous force production, which is often required in sports. A new test, the one-leg speedy test, was designed for additional demands. This test required knee joint stability in multiple planes and directions. In addition, a dynamic balance ability and coordination of the whole body is required to maintain balance. Interestingly, when the hop test demanded an increased endurance and more complex task, the difference in the LSI between the dominant and non-dominant legs decreased to 101–104 %. These results may indicate that fatigue assimilates the differences in leg dominance. In addition, the specificity of this test showed more acceptable values, between 86 and 95 %, and was higher than that of the other one-leg tests. The importance of quick movements in a fatigued state has been emphasized in relation to the prevention of ACL injuries [28]. Therefore, we administered the quick feet test as the last test in the battery. In addition to speed, the test also requires a high degree of concentration to avoid mistakes. These physical requirements are important for competitive sports. Furthermore, neuromuscular control and good coordination have also been documented to decrease the likelihood of knee injuries [16]. Despite the importance of coordination and balance in knee injury prevention, the results of one-leg stability tests have not yet been correlated with return to sport. The stability test on the MFT Challenge Disc requires balance and coordination in both two- and one-legged performance. The comparison between the dominant and non-dominant legs revealed an LSI of 98 %, indicating a poor performance in the assumed dominant leg. The selection of the dominant leg in this study was based on the subjective dominance of fundamental movements. However, the definition of the dominant leg is not clear. According to Kimura and Asaeda [18] as well as Miyaguchi and Demura [20], the ball kicking movement showed marked right-leg dominance. In the present study, the right leg was declared as the dominant leg for most of the participants. However, with regard to return to sport, it needs to be considered that both legs are used equally in some sports (e.g. running, alpine skiing, swimming) and that other sports require a specific leg (e.g. jumping events in track and field, ball games). In practice, most of the functional tests are individual tests. Functional movement tests within a test battery were utilized as an outcome measure or a measurement of function [22]. Myer et al. [22] tested functional deficits in athletes following ACL rupture compared with a healthy control group. The low number of participants in the ACL group (18) may limit the results. Narducci et al. [23] stated that none of the clinicians utilized functional performance tests as a measure of readiness to return to sport. The present study collected information from more than 400 healthy subjects that allows for the creation of normative data. These data serve as a basis for the classification of the potential of the knee during the rehabilitation process and, therefore, evidence for use in decision-making regarding an unrestricted return to activity. To represent age- and population-based ACL injuries, four age categories according to gender were chosen to provide comprehensive normative data. We tested active subjects to generate normative data because most ACL ruptures appear in athletic individuals who want to become active again. The present test battery can now be used in the day-by-day clinical work to draw conclusions regarding its ability to assist in decision-making regarding returning to sport. Based on the normative data, the test results of an ACL-reconstructed patient within the rehabilitation period can now be objectively evaluated. Only an at least functionally good outcome in each subtest should be reached to minimize the risk of a re-ruptures of the ACL. There are some limitations that need to be considered for further studies. The present test battery includes seven subtests to asses general sports performance. However, there is often a large discrepancy between clinical outcomes and the rate of successful return to high-level sport. Therefore, more demanding criteria and functional tests need to be established to determine sport-specific return-to-sport outcomes in high-level competitive athletes [4]. Overall, it must be considered that the test battery facilitates the attainment of information regarding the actual functional status of the knee; however, in addition to physical parameters, the psychological status of an athlete who experienced an ACL injury is of great importance for guaranteeing a successful return to sport. Thus, in addition to the functional test battery, psychological outcome measures must be added to ensure that participants in sports are physically and psychologically capable of returning to sport [12].

## Conclusion

The present study features normative data from seven functional performance tests and, thus, aids in the return-to-sport timeline by identifying functional deficits of the knee in clinical work. At this point, it needs to be considered that limb asymmetries of more than 10 % were evident in healthy subjects within the one-leg hop testing. This aspect will be discussed in the second part of the study, where the clinical utility of the test battery will be explained. It can be concluded that all tests are simple to administer, and the application of quick on-field tests that do not require sophisticated and expensive devices simplifies testing during the rehabilitation period.
